# Physiology of the Secretion of Sweat

**Published:** 1879-08

**Authors:** 


### PHYSIOLOGY OF THE SECRETION OF SWEAT.

That the secretion of sweat is under the control of the nervous system has been recognized for some years past, Luchsinger and others having demonstrated that a copious discharge of this secretion can be induced in the feet of the cat and dog by stimu'ation of sciatic and brachial nerves. That it is essentially independent of any changes in the circulatory system is shown by the fact that it can be made to occur in an amputated member, and in limbs the temperature of which is below the normal. The secretion is thus shown not to be a mere transudation, but the result of the activity of special glandular cells, called forth, as in the case of the salivary glands, by the stimulation of certain nerves. The sudoriparous nerve, running in the sciatic nerve, is derived from the abdominal cord of the sympathetic; for if this be divided, and the lower extremity be stimulated, perspiration breaks out on the hind foot, though if the sciatic be first divided, no such secretion is observed. After division of the sympathetic in the abdomen on one side, the animal no longer sweats on that side when exposed to heat. But the fibres do not rise in the great sympathetic; they appear to emerge from the spinal cord by the rami communicans of the first four lumbar roots, and the last two or three dorsal. Sweating can be induced by reflex action, and also in a very marked and singular manner by jaborandi, and by the active principle of that drug—pilocarpin. In from three to five minutes after the subcutaneous injection of a solution of hydrochlorate of pilocarpin, in a man, the flow of saliva increases, perspiration appears, first on the head, and then gradually over the whole body, and lasts about an hour, or, if the patient be in bed, for two or even three hours. This effect Luchsinger considers to be due to the pilocarpin acting as a direct stimulant to the nerve-centres. He tied the abdominal aorta in a cat, and then injected pilocarpin into a vein. Under these conditions the pilocarpin was unable to reach the glands in the posterior extremities, and thus to act as a direct stimulant; nevertheless the feet were soon bathed in sweat. Atropin inhibits the secretion of sweat, for if, after the injection of one-hundreth of a gramme of pilocarpin, three one-thousanths of a gramme of atropin be injected, the commencing perspiration is arrested in about ten minutes. If now a hundreth of a grain of pilocarpin be injected into one of the feet, beads of sweat burst forth on this foot; but the rest of the body, being still under the influence of atropin, remains dry. The experiments of Luchsinger have been repeated and confirmed by Nawrocki, who satisfied himself that there is a common centre in the medulla for the secretion of sweat in both fore and hind feet. He followed the course of the fibres innervating the glands of the fore limb, and ascertained by means of sections at different points that they leave the spinal cord between the third and fifth cervical vertebræ. These fibres enter the brachial plexus with the thoracic portion of the sympathetic, and are occasionally confined within the sheath of the median nerve, though more frequently they are distributed between the median and ulnar nerves, the median having the larger share. Adamkiewiez, in a more recent publication, finds, like his predecessors, that the secretion of sweat is independent of the circulation, and that it may be induced by artificial or voluntary stimulation of the muscles, or of their nerves, by mental stimuli, as by the imagination; and, lastly, as a reflex act by stimuli applied to the skin. In man the secretion is always bilateral and symmetrical, and is not necessarily eliminated in the immediate vicinity of the point stimulated. Heat excites it, and, indeed, the activity of the secretion seems to stand in direct relation with the temperature of the several parts of the body. His views differ from those of Luchsinger in regard to the nervous apparatus, for he believes that the motor centre of the secretion is situated on the surface of the brain. The nerves pass through the medulla to the spinal cord, and unite at the secretory centres, which are probably placed in the anterior horns of the gray matter. From these horns secretory fibres emanate and leave the cord in connection with motor nerves, whilst others enter the sympathetic at higher points of the cord. Vulpian, in following out these experiments, found that although some of the excito-sudoral fibres may, in the cat, pass from the sympathetic to the sciatic nerve, yet that there are others in considerable number which pass directly from the spinal cord by the seventh lumbar and first sacral—that is to say, by the roots of the sciatic itself. Vulpian points out that an interesting parallel may thus be drawn between the nervous mechanism of the sweat glands and that of the salivary glands; for it is known that the submaxillary glands receive excito-salivary fibres from the chorda tympani, and other fibres from the cervical portion of the great sympathetic. He is, however, unable to coincide in the view that the filaments for the sweat-glands in the foot and fore-limb of the cat pass out from the cord *entirely* with the spinal roots of the superior thoracic ganglion. A large part no doubt do so, but others accompany the roots of the spinal nerves entering into the formation of the brachial plexus. Luchsinger and Truempy have quite recently investigated the chemical properties of sweat. An acid reaction is generally attributed to this secretion, but these observers have ascertained that in man as well as in the cat the reaction is really alkaline, and that the acidity which has been observed is due to the fact that the secretion of the sebaceous glands is ordinarily acid, or rather becomes so in the act of decomposition, to which it is prone. The whole subject has been well analyzed and treated by M. Blanchard in the *Progres Medicale.—Lancet, June* 14, 1879.

